# Supplementary material for: In Vivo Study of the Efficacy and Safety of 5-Aminolevulinic Radiodynamic Therapy for Glioblastoma Fractionated Radiotherapy
Source: Int J Mol Sci. 2021 Sep 9;22(18):9762. doi: 10.3390/ijms22189762 (PMC8470662; doi:10.3390/ijms22189762)
Supplement: Supplementary file 1 [file ijms-22-09762-s001.zip › ijms-1339716-supplementary.pdf]

International Journal of
Molecular Sciences

MDPI

Supplementary materials

Table S1. Body weight of mice transplanted U251MG.

Group	0 Week	1 Week	2 Weeks	3 Weeks	4 Weeks	5 Weeks	6 Weeks	Final
NT	20.2 ± 0.9	20.4 ± 1.0	21.1 ± 0.6	21.6 ± 1.1	-	-		
120ALAT	20.8 ± 0.8	20.9 ± 0.8	22.0 ± 0.8	21.9 ± 1.2	-	-		
XT	20.6 ± 1.4	20.7 ± 1.3	22.1 ± 1.6	21.9 ± 1.1	22.0 ± 1.2	23.2 ± 1.3	23.0 ± 1.1	22.1 ± 0.7
60ALAXT	20.7 ± 0.9	20.8 ± 0.9	22.0 ± 0.8	22.1 ± 0.2	21.7 ± 1.0	23.0 ± 1.0	22.6 ± 0.9	22.7 ± 1.0
120ALAXT	19.8 ± 0.8	20.0± 0.3	21.1 ± 1.1	20.9 ± 0.9	21.1 ± 0.9	22.3 ± 0.9	22.2 ± 0.9	22.0 ± 1.2

Data are presented as means ± standard deviations.

Table S2. Body weight of mice transplanted U87MG.

Group	0 Week	1 Week	2 Weeks	3 Weeks	4 Weeks	5 Weeks	6 Weeks	7 Weeks
NT	20.5 ± 0.6	20.6 ± 0.5	20.3 ± 1.3	20.6 ± 1.1		-	-	-
120ALAT	20.6 ± 0.9	20.3 ± 0.7	20.5 ± 1.3	20.4 ± 0.7		-	-	-
XT	21.4 ± 0.7	21.0 ± 0.2	21.4 ± 1.0	21.2 ± 0.7	21.2 ± 0.5	20.7 ± 0.8	20.4 ± 1.1	19.7 ± 1.0
60ALAXT	21.9 ± 0.8	21.8 ± 0.6	22.2 ± 0.9	22.3 ± 0.9	22.4 ± 1.1	21.7 ± 0.9	22.7 ± 1.1	21.7 ± 0.7
120ALAXT	21.2 ± 0.6	20.7 ± 0.8	21.9 ± 0.8	21.2 ± 1.1	20.9 ± 0.9	20.7 ± 0.9	21.3 ± 0.9	19.6 ± 1.0

Group	8 Weeks	9 Weeks	10 Weeks	Final
NT	-	-	-	-
120ALAT	-	-	-	-
XT	19.4 ± 1.2	19.8 ± 1.3	19.5 ± 1.5	18.9 ± 1.3
60ALAXT	21.2 ± 1.1	21.0 ± 1.4	20.5 ± 1.6	18.4 ± 1.2
120ALAXT	19.9 ± 0.6	19.5 ± 0.5	19.3 ± 0.6	18.6 ± 0.8

Data are presented as means ± standard deviations.

Table S3. Body weight of mice for safety test.

Group	0 week	1 week	2 weeks	3 weeks	4 weeks	5 weeks	6 weeks	final
NT	19.7 ± 0.6	20.7 ± 0.5	22.1 ± 0.3	23.2 ± 0.4	23.9 ± 0.5	24.1 ± 0.6	25.0 ± 0.8	25.5 ± 0.9
240ALAT	20.7 ± 0.6	20.7 ± 1.0	21.9 ± 0.8	22.4 ± 0.9	22.7 ± 1.1	23.4 ± 0.7	23.9 ± 0.7	24.1 ± 1.0
XT	20.5 ± 0.5	19.7 ± 0.9	19.7 ± 0.5	19.5 ± 0.5**	19.5 ± 0.6**	18.1 ± 0.6**	18.6 ± 0.9**	17.6 ± 0.9**
120ALAXT	20.9 ± 0.6	20.5 ± 0.8	20.3 ± 0.4	21.1 ± 0.7**	20.1 ± 0.5**	19.2 ± 0.5**	20.4 ± 0.4**	19.1 ± 1.0**
240ALAXT	20.1 ± 0.1	19.6 ± 0.4	20.3 ± 0.3	20.6 ± 0.5**	20.0 ± 0.6**	19.0 ± 0.7**	20.1 ± 0.6**	19.5 ± 0.5**

Data are presented as means ± standard deviations. Statistical significance in a one way ANOVA and Turkey post-test relative to the experiment performed at without irradiation at the same time is indicated by (*$p < 0.05$, **$p < 0.01$).

Table S4. Serum biochemical test results.

Group	TP (g/dL)	ALB (g/dL)	A/G	BUN (mg/dL)	CRE (mg/dL)	Na (mEq/L)
NT	4.90 ± 0.52	3.30 ± 0.37	2.08 ± 0.18	31.1 ± 3.0	0.13 ± 0.02	150.3 ± 3.8
240ALAT	4.40 ± 0.27	2.90 ± 0.12	1.95 ± 0.17	29.7 ± 4.6	0.13 ± 0.01	146.3 ± 1.8
XT	4.22 ± 0.29	2.86 ± 0.16	2.28 ± 0.60	24.7 ± 3.7 *	0.13 ± 0.02	148.4 ± 1.4
120ALAXT	4.06 ± 0.16 *	2.88 ± 0.12	2.44 ± 0.12	21.9 ± 3.0 **	0.13 ± 0.02	146.6 ± 0.8
240ALAXT	4.30 ± 0.36	2.94 ± 0.14	2.18 ± 0.20	20.9 ± 3.1 **	0.13 ± 0.02	146.8 ± 0.7

Group	K (mEq/L)	Ca (mg/dL)	IP (mg/dL)	AST (IU/L)	ALT (IU/L)	ALP (IU/L)
NT	6.73 ± 1.69	9.45 ± 0.15	7.05 ± 0.75	304.3 ± 80.9	71.5 ± 20.8	350.0 ± 39.3
240ALAT	6.98 ± 1.31	9.08 ± 0.29	7.23 ± 0.29	314.2 ± 63.6	109.8 ± 40.3	372.8 ± 28.1
XT	6.72 ± 0.70	9.08 ± 0.21	4.70 ± 0.32 **	207.6 ± 62.2	65.4 ± 24.4	170.0 ± 20.2 **
120ALAXT	6.86 ± 0.51	8.82 ± 0.10 **	5.72 ± 0.47 *	245.2 ± 43.1	81.6 ± 24.3	188.6 ± 20.0 **
240ALAXT	7.12 ± 1.15	8.98 ± 0.23 *	7.08 ± 0.53	408.4 ± 153.8	174.0 ± 72.8*	269.2 ± 46.2 *

Group	T-CHO (mg/dL)	TG (mg/dL)	T-BIL (mg/dL)	GLU (mg/dL)
NT	79.8 ± 7.7	147.3 ± 29.9	0.05 ± 0.05	215.8 ± 69.8
240ALAT	93.3 ± 4.5	146.0 ± 42.0	0.08 ± 0.02	233.8 ± 47.6
XT	84.4 ± 12.7	92.0 ± 29.8	0.05 ± 0.02	177.0 ± 41.8
120ALAXT	86.2 ± 7.1	69.6 ± 16.9 *	0.07 ± 0.02	231.8 ± 25.8
240ALAXT	102.2 ± 14.6	110.2 ± 43.3	0.06 ± 0.01	190.6 ± 30.1

Data are presented as means ± standard deviations. Statistical significance in a one way ANOVA and Turkey post-test relative to the experiment performed at without irradiation at the same time is indicated by (*$p < 0.05$, **$p < 0.01$).
